# Health literacy and glycemic control in patients with diabetes: a tertiary care center study in Brazil

**DOI:** 10.1186/s13098-020-0519-6

**Published:** 2020-02-03

**Authors:** Marilia B. Gomes, Luiza Harcar Muniz, Laura Gomes Nunes Melo, Marcela Haas Pizarro, Bianca Senger Vasconcelos Barros, Deborah Conte Santos, Carlos Antonio Negrato

**Affiliations:** 1 Diabetes Unit, Department of Internal Medicine, State University Hospital of Rio de Janeiro, Rio de Janeiro, Brazil; 2 Medical Doctor Program, University of São Paulo-School of Dentistry, Bauru, São Paulo, Brazil

**Keywords:** Type 1 diabetes, Type 2 diabetes, Glycemic control, Health literacy, Diabetes management

## Abstract

**Background:**

The primary objective of our study was to determine which factors influence health literacy (HL) in patients with type 1 diabetes (T1D) and type 2 diabetes (T2D), and the secondary one was to evaluate the influence of HL on glycemic control.

**Methods:**

This was an observational, cross-sectional study with 347 patients (144 with T1D and 203 with T2D), conducted between December 2014/December 2017. Data were obtained from medical records and/or questionnaire. The short test of Functional Health Literacy (S-TOFHLA) was used to evaluate HL.

**Results:**

Age and years of school attendance were the most important variables associated with better performance in S-TOFHLA mainly in patients with T1D. A correlation between age and years of school attendance with S-TOFHLA score was observed in both groups of patients. After an unadjusted analysis, more patients with T1D presented adequate HL [119 (82.6%) vs 87 (44.8%, p < 0.001)]. Patients with T1D had higher scores than patients with T2D (84.4 ± 21.4 vs 61.6 ± 26.8 points, p < 0.001), respectively. This difference did not persist after adjustment for age and years of school attendance (73.04 ± 2.14 ± vs 70.04 ± 1.76 points) respectively, p = 0.348). No difference was found in HbA1c levels according to S-TOFHLA. All patients with T1D and HbA1c levels < 7.0% (53 mmol/mol) had adequate HL.

**Conclusions:**

A considerable number of patients with either T1D or T2D did not have adequate HL. Overall, age and years of school attendance were the most important variables associated with better performance of S-TOFHLA. Although no difference was found in HbA1c levels according to S-TOFHLA, patients with T1D who self-reported as White, with more years of school attendance, and higher HL score reached more frequently a good glycemic control. Finally, in addition to therapeutic regimens, approaches on diabetes management should also include patients’ HL evaluation along with psychological and social aspects.

## Background

Diabetes is a chronic disease with a prevalence that varies widely throughout the world and is continuously increasing [1, 2]. Nowadays, intensive diabetes management including insulin therapeutic regimens or therapies with oral agents, self-monitoring of blood glucose (SMBG), practice of regular physical activity and medical nutrition therapy are the basis of treatment for patients with either type 1 diabetes (T1D) or type 2 diabetes (T2D) [3]. These therapies are important to obtain and keep a satisfactory glycemic control and avoid diabetes-related chronic complications that result in very high direct and indirect costs and are associated with high rates of mortality [4, 5]. Ever since the results of the UKPDS [6] and the Diabetes Control and Complications Trial (DCCT) and Epidemiology of Diabetes Interventions and Complications (EDIC) studies were brought up to light, showing that good glycemic control reduces the risk of diabetes-related chronic complications [7], different intensive therapies were proposed for both groups of patients [8, 9]. However, to reach and maintain a good glycemic control is still a challenge in clinical practice, mainly among minorities [10]. In the US, the Exchange study has shown that only 64% and 43% of children and adolescents, met the glycemic control targets, respectively [11], while in Europe, the Eurodiab study has shown that only 16% of their patients had an adequate glycemic control [12]. Considering patients with T2D, the Discover study, an observational worldwide survey, showed that the majority of the patients were out of good glycemic control targets [13]. In previous Brazilian studies, only 13.2% of patients with T1D [14] and less than 50% of patients with T2D presented HbA1c at goal [15].

Moreover, many other factors can contribute to poor glycemic control in daily clinical practice such as poor or absent family support, social economic inequalities, poor continuous educational support regarding many aspects of diabetes as well as its management, inadequate literacy and cognitive dysfunction due to hypoglycemia or chronic hyperglycemia [16–20]. Health literacy (HL) and cognitive function should then be considered cornerstone tools for an adequate management of all chronic diseases including diabetes [21, 22].

In Brazil, literacy is still a cumbersome problem. According to PNAD (Pesquisa Nacional por Amostra de Domicílios or National Household Sample Survey), 11.8 million (7.2%) of Brazilians could be considered as illiterate [23]. Besides this, data from INAF (Indicador de Alfabetismo Funcional or Functional Literacy Indicator), evaluating the literate population, showed that about 27% were considered to be non-functionally literate [24]. Recently, the term HL was introduced in educational medical health programs and is defined as the ability to use any type of health information to take appropriate decisions and follow correctly treatments related to any type of disease [21]. Nowadays, HL is an important issue in diabetes management due to its high level of complexity [25, 26]. In a recent review, the relationship between HL and diabetes included many aspects that are important for the management of the disease such as listening and speaking, writing and reading, and also the ability to understand and use numbers [25, 26]. Although all the above mentioned skills could be indirectly related to glycemic control as well as to the evolution of diabetes-related chronic complications, the results of some studies are still controversial because the best tool to evaluate HL, as well as the need to perform it in routine clinical care, are still undefined [26–38].

The test of Functional Health Literacy (TOFHLA) is commonly used in medical research in several countries, including Brazil, and has been considered a suitable tool to evaluate HL [27]. This test has three versions: the complete version, a 67-item, 22-min test evaluating reading comprehension and numeracy skills; the short version of TOFHLA (S-TOFHLA), a 40-item, 12-min test of numeracy skills (4-items) and reading and comprehension (36-items) [27]. According to the score obtained from TOFHLA, patients are classified as illiterate, literally inadequate or literate [27]. However, in developing countries in which a large number of people are still non-functionally literate, such as Brazil, it is important to establish the relationship between HL, educational level and some aspects of chronic diseases that could be relevant for its morbidity [21, 39]. The TOFHLA comprises questions related to self-efficacy, that is an important tool for diabetes management that is related to glycemic control.

The above mentioned factors could be considered barriers, sometimes invisible for diabetes teams, hindering the achievement of good glycemic control in daily routine clinical practice and thus, increasing the risk for diabetes-related chronic complications.

The primary aim of our study was to determine which factors may influence HL in patients with diabetes, and the secondary one, to verify the influence of HL on glycemic control.

## Research design, study participants and methods

This was an observational, cross-sectional study, conducted at the Diabetes Unit from Rio de Janeiro’s State University (UERJ) between December 2014 and December 2017. All consecutive patients with T1D or T2D in use of oral drugs as well as under insulin therapy, attending the Diabetes Unit who agreed to participate, were enrolled in the study. This study was approved by the ethics committee of Hospital Universitário Pedro Ernesto. Written informed consent was obtained from all patients. The inclusion criteria were patients diagnosed with either T1D or T2D according to ADA’s criteria [3], aged 19 to 80 years, followed at the outpatient clinic for at least 6 months. The exclusion criteria were the presence of cerebrovascular disease, pregnancy, history of acute infection in the previous 3 months, severe hypoglycemia or diabetic ketoacidosis in the last month, blindness, mental health disease and any disease that could interfere with the accuracy of the answers.

The following variables were assessed using a questionnaire during a clinical visit: current age, age at diagnosis, diabetes duration, years of school attendance, economic status, self-reported color-race, systolic and diastolic blood pressure, antihyperglycemic therapeutic regimens, use of statins and anti-hypertensive drugs, smoking and drinking habits. Body mass index (BMI) was determined by dividing an individual’s weight (kg) by the square of the height (m^2^).

We have adopted the ADA’s goals for adequate metabolic and clinical control: good glycemic control was defined as HbA1c < 7% (53 mmol/mol) [40]. Poor glycemic control was arbitrarily defined as HbA1c ≥ 9% (75 mmol/mol). HbA1c, creatinine, triglycerides, total cholesterol, high-density lipoprotein (HDL) cholesterol, low-density lipoprotein (LDL) cholesterol levels were measured during the clinical visit or were obtained from the patients’ medical records. HbA1c was measured using high-performance liquid chromatography (HPLC, Bio-Rad Laboratories, Hercules, California, USA). Creatinine, HDL cholesterol, total cholesterol, and triglycerides were measured using enzymatic techniques. Smoking was defined as the current use of more than one cigarette per day at the time of the interview and drinking was defined according to the guidelines of the World Health Organization (WHO) [41].

## Measurement of health literacy

The evaluation of HL was carried out by a trained researcher for the application of the used instruments in an adequate room with attenuation of external noise at Diabetes Unit. The following tests were applied to evaluate HL: the short version of TOFHLA (S-TOFHLA), a 40-item, 12-min test of numeracy skills (4-item, 28 points) and reading and comprehension (36-item, 72 points) [42]. Results for S-TOFHLA, which maximum score was 100 points (1 point for each correct answer), were categorized into illiteracy (0–53 correct answers); inadequate literacy (54–66 correct answers) and adequate literacy (67–100 correct answers) [42]. S-TOFHLA tests have been used in Portuguese language [33]. The tests were performed exclusively in patients with capillary glycemia (CG) ≥ 100 mg/dl before the beginning of the tests. Patients with CG < 100 mg/dl were instructed to eat a light meal with carbohydrates and wait for a re-test of CG to proceed with the evaluation or had the evaluation remarked. At this time we also tested the visual acuity by the Rosenbaum Pocket Vision Screener test [43]. Patients with abnormal visual acuity were tested for card reading and, when considered able, performed the tests.

### Economic status definition

Economic status was defined according to the Brazilian Economic Classification Criteria, which also categorize education as illiterate/incomplete primary education, complete primary education/incomplete secondary education, complete secondary education/incomplete high school, complete high school/some college or complete college education [22]. The following classes of economic status were considered: high, middle, low and very low.

## Statistical analysis

Firstly, an exploratory analysis was carried out. Data are presented as the means (±SD) or mean ± standard error (SE) for ANCOVA data or medians (minimum–maximum) for continuous variables and numbers (relative frequencies) for discrete variables. Comparisons between independent continuous variables were performed using independent, two-sided *t*-tests or Mann–Whitney when indicated. ANOVA with Zidak correction, was used as indicated. Two-sided Z-tests were used for comparisons between discrete variables. Bivariate Pearson correlation was performed between S-TOFHLA score as continuous variable and demographic and laboratorial data with further analysis adjusted for years of school attendance (partial correlation). Forward Wald stepwise multivariate logistic regression analysis was performed with HbA1c < 7.0% (53 mmol/mol) and HbA1c ≥ 9.0% (75 mmol/mol) as the dependent (outcome) variables. Other predictive variables, such as self-reported color-race, age, years of school attendance, gender and duration of diabetes were controlled in the analysis. In this latter model we added S-TOFHLA separately from years of school attendance due to its interaction. ANCOVA was performed with total S-TOFHLA score as dependent variable and with age and years of school attendance as covariates for adjustment. Multivariate logistic analysis was also performed with S-TOFHLA adequate and inadequate as dependent variable and type of diabetes, duration of diabetes, self-reported color-race, age and years of school attendance as independent variables. In these models the Nagelkerke R-squared value was also calculated. Analysis were performed using SPSS version 17.0 (SPSS, Inc., Chicago, Illinois). Odds ratios with 95% CIs were expressed as indicated. A two-sided *p* value less than 0.05 was considered significant.

## Results

### Overview of the demographic and clinical data of the studied population

Table 1 shows the clinical and demographic data of the studied population. Only 5 (3.7%) and 4 (2.0%) patients with T1D and T2D, presented CG < 100 mg/dl, respectively, but no one presented symptoms of hypoglycemia. These patients were re-tested for CG, and when the results were over 100 mg/dl, they were evaluated. All the patients with T1D had visual acuity considered adequate to perform the test (S-TOFHLA), but five (2.5%) patients with T2D presented an abnormal visual acuity. These patients did not perform the test.

**Table 1:** Demographic, clinical and laboratory data of the studied population

Variables	T1D	T2D
Age, years	37.5 ± 12.5	59.7 ± 10.4
Age, n (%) (years)		
< 25	29 (20.1)	2(1.0)
25–44	67 (46.5)	16(7.9)
45–64	46 (31.9)	123 (60.6)
≥ 65	2 (1.0)	62 (30.5)
Gender, n (%)		
Male	63 (43.7)	71(37.9)
Female	81 (56.3)	132 (65)
Self-reported color-race, n (%)^a^		
White	73 (50.7)	77(37.9)
Non-White	71 (49.3)	126(62.1)
Years of school attendance	12.4 ± 3.1	8.4 ± 4.4
Economic status^b^		
Low	95 (66.0)	123 (60.6)
Very low	10 (6.9)	34 (16.7)
Medium	31 (21.5)	18 (8.9)
BMI (kg/m^2^)	24.6 ± 4.2	29.9 ± 5.2
Initial CG (mg/dl)	195.8 ± 81.4	184.1 ± 64.8
Final CG (mg/dl)	172.7 ± 89.7	174.1 ± 70.6
HbA1c (%)	8.9 ± 1.9	8.1 ± 3.2
< 7.0 n (%)	16 (11.8)	56(26.6)
≥ 9.0 n (%)	63(46.3)	60(29.6)
HbA1c, mmol/mol	74.5 ± 21.0	65.3 ± 19.5
Smokers, years; n (%)	15 (7.5)	33 (16.2)
Drinkers, years; n (%)	25 (17.4)	44(21.6)
Diabetes treatment; years, n (%)		
Oral drugs^c^	48 (23.9)	190 (93.5)
Antihypertensives	40 (27.8)	130(64.0)
Insulin	144 (100.0)	92(45.3)
Statins	35 (24.3)	124(61.1)

*T1D* Type 1 diabetes, *T2D* Type 2 diabetes. Data are presented as n (%) or means (standard deviation), *BMI* body mass index, *CG* capillary glycemia

^a^Non-White (Mulattos and Blacks)

^b^Missing: 8 (5.6%) for T1D and 28 (13.8%) for T2D

^c^The oral drug used by patiens with T1D was metformin

### Demographic, clinical and laboratory data of the studied population stratified according to S-TOFHLA

All patients with either T1D (n = 5; 3.5%) and T2D (n = 24; 12.4%) with inadequate literacy S-TOFHLA were considered as having illiteracy in S-TOFHLA. Patients with T1D had higher scores than patients with T2D (84.4 ± 21.4 vs 61.6 ± 26.8 points, p < 0.001), respectively. This difference did not persist after adjustment for age and years of school attendance (73.04 ± 2.14 ± vs 70.04 ± 1.76 points) respectively, p = 0.348. The unadjusted time required to complete the S-TOFHLA was longer in patients with T2D compared to patients with T1D (9.4 ± 1.8 vs 7.7 ± 2.1 min, p < 0.001), respectively. This difference did not persist after adjustment for age and years of school attendance (8.75 ± 0.17 vs 8.67 ± 0.14, p = 0.757), respectively. Unadjusted analysis showed that more patients with T1D were considered literate than patients with T2D [119 (82.6%) vs 87 (44.8%), p < 0.001], respectively, and have reached the maximum score of 100 points than patients with T2D [51 (79.7%) vs 13 (20.3%), p < 0.001], respectively.

Considering the part of S-TOFHLA which evaluated numeracy skills, patients with T1D had higher scores than patients with T2D (23.0 ± 7.2 vs 20.3 ± 7.6 points; p = 0.001), respectively. More patients with T1D reached the maximum score of 28 points than patients with T2D [84 (58. 3%) vs 69 (34.0%); p < 0.001], respectively. The time required to complete this part of S-TOFHLA was longer in patients with T2D compared to patients with T1D (2.9 ± 1.2 vs 2.3 ± 0.9 min, p < 0.001), respectively. Considering the part of S-TOFHLA which evaluated reading and comprehension, patients with T1D had a higher score than patients with T2D (61.4 ± 16.1 vs 41.3 ± 21.8 points; p < 0.001), respectively. More patients with T1D reached the maximum score of 72 points than patients with T2D, [66 (45.8%) vs 21 (10.3%); p < 0.001], respectively. In patients with T1D the total score of S-TOFHLA was not associated with gender and self-reported color-race but in patients with T2D a higher score of S-TOFHLA was observed in patients who self-reported as White (70.9 ± 24.1 vs 55.9 ± 27.1; p < 0.001), respectively. No association with gender was noted in patients with T2D. No difference was noted in the average HbA1c in patients with either T1D or T2D, either literate or illiterate. The demographic, clinical and laboratory data (glycemic control) stratified according to S-TOFHLA are described in Table 2.

**Table 2:** Demographic, clinical and laboratory data stratified according to S-TOFHLA

S-TOFHLA			
Variables	Literate	Illiterate	p-value
T1D			
	N = 119 (82.6%)	N = 25 (17.4%)	
Age, years	36.5 ± 12.3	42.2 ± 12.1	0.04
Gender, F, n (%)	70 (86.4)	11 (13.6)	0.17
Duration of diabetes, years	18.3 ± 10.2	19.0 ± 10.0	0.70
Self-reported color-race, n (%)			0.80
White	61 (83.6)	12 (16.4)	
Non White^a^	58 (81.7)	13 (18.3)	
Years of study	13.1 ± 2.8	9.3 ± 2.9	< 0.001
HbA1c (%)	8.9 ± 1.9	9.3 ± 1.6	0.30
HbA1c (mmol/mol)	73.8 ± 21.7	77.9 ± 17.5	
T2D^b^			
	N = 87 (42.9)	N = 107 (52.7)	
Age, years	55.9 ± 10.6	62.2 ± 9.6	< 0.001
Gender, F, n (%)	59 (46.5)	68 (53.5)	0.30
Duration of diabetes, years	11.8 ± 7.5	16.5 ± 9.7	< 0.001
Self-reported color-race, n (%)			< 0.001
White	41 (56.2)	32 (43.8)	
Non White	46 (38.0)	75 (62)	
Years of study	10.9 ± 3.9	7.1 ± 3.7	< 0.001
HbA1c	8.3 ± 1.9	8.0 ± 1.7	0.15
HbA1c (mmol/mol)	67.7 ± 20.8	63.6 ± 18.7	

*T1D* type 1 diabetes, *T2D* type 2 diabetes

^a^Non-White (Mulattos and Blacks)

^b^Five patients were excluded from S-TOFHLA for inadequate visual acuity and four patients missed their appointment

The number and proportion of patients who answered correctly each question of both domains of S-TOFHLA are described in Table 3. The time required to complete this part of S-TOFHLA was longer in patients with T2D compared with patients with T1D (6.6 ± 0.9 vs 5.4 ± 1.5 min, p < 0.001), respectively. More patients with T1D with adequate HL had HbA1c levels < 7.0% (53 mmol/mol) than patients with inadequate HL [16 (100%) vs 49 (77.8%); p = 0.038], respectively. These patients had a higher score in TOFHLA, (94.7 ± 8.2 vs 81.5 ± 24.2, p = 0.03), respectively.

**Table 3:** Number and proportion of patients who answered correctly the domains of the S-TOFHLA test

Variables Numeracy items:	T1D	T2D
	N = 144	N = 194
Table medication every 6 h	115 (79.9)	139 (71.6)
Normal blood sugar	116 (80.6)	162 (83.5)
Appointment	123 (85.4)	120 (61.9)
Table medication before lunch	120 (83.3)	141 (72.7)
Reading comprehension (points)		
0–18	3 (2.1)	37 (19.1)
19–36	15 (10.4)	51 (26.3)
37–55	20 (13.9)	42 (21.6)
56–72	106 (73.6)	64 (33.0)

### Correlation between total S-TOFHLA score and demographic, clinical and laboratory data

Considering the group of patients with T1D, a correlation was observed between S-TOFHLA score and age (r = − 0.24; p = 0.004), age at diagnosis (r = − 0.22; p = 0.006) and years of school attendance (r = 0.51; P < 0.001). The partial correlation coefficient between age and S-TOFHLA score persisted when controlling for the effect of years of school attendance (r = − 0.176; p = 0.04) but did not persist for age at diagnosis. No correlation was observed with duration of diabetes and HbA1c levels. No correlation was noted between the score of S-TOFHLA test domains (numeracy skills, reading and comprehension) with HbA1c levels and CG at the beginning and at the end of the test.

Considering the group of patients with T2D, a correlation was observed between S-TOFHLA score with age (r = − 0.33; p = 0.004), and with years of school attendance (r = 0,53; p < 0001). The partial correlation coefficient between age and S-TOFHLA score persisted when controlling for the effect of years of school attendance (r = − 0.22; p = 0.01). No correlation was observed with duration of diabetes and HbA1c levels. No correlation was also noted between the score of S-TOFHLA domains (numeracy skills, reading and comprehension) and HbA1c levels.

### Multivariate analysis with S-TOFHLA adequate and inadequate literacy as dependent variable

Multivariate analysis performed with adequate and inadequate literacy S-TOFHLA as dependent variables, showed that all the independent variables which entered in the model even after adjustment could explain only 45.5% (Nagelkerke R-squared) of a given patient reaching an inadequate S-TOFHLA. The independent variables associated with inadequate S-TOFHLA were lower years of study [B = − 0.282;OR = 0.754 (0.696–0.817), p < 0.001] and higher age [B = 0.043;OR = 1.044 CI 95% (1.022–1.067 p < 0.001)]. Type of diabetes, self-reported color-race and duration of diabetes did not reach statistical significance.

### Multivariate analysis in patients with type 1 diabetes with HbA1c at goal as the dependent variable

Multivariate analysis performed with HbA1c at goal and HbA1c ≥ 9.0% (75 mmol/mol) as dependent variable, showed that all the independent variables which entered in the model even after adjustment could explain only 21.3% (Nagelkerke R-squared) of a given patient reaching the goal for HbA1c. In the model with S-TOFHLA as independent variable, HbA1c at goal was associated with White self-reported color-race [B = 1.207; OR = 3.354 CI 95% (1.004–11.205), p = 0.049] and had a tendency to be associated with HL [B = 0.055;OR = 1.057 CI 95% (0.99–1.123), p = 0.07]. In the model with years of school attendance as independent variable, HbA1c at goal was associated with years of school attendance [B = − 0.26, OR = 0.771 (0.623–0.995), p = 0.017].

### Multivariate analysis in patients with type 2 diabetes with HbA1c at goal as the dependent variable

Multivariate analysis performed with HbA1c at goal and HbA1c ≥ 9.0% (75 mmol/mol) as dependent variables, showed that no independent variables which entered in the model, even after adjustment, reached statistical significance or a tendency.

## Discussion

Our study, conducted in a tertiary care center, showed that a considerable number of patients with either T1D or T2D did not have adequate HL. Our results have shown that the independent variables associated with inadequate S-TOFHLA were lower years of school attendance and higher age. However, after adjustment for age and years of school attendance, type of diabetes, self-reported color-race and duration of diabetes did not reach statistical significance. S-TOFHLA score was related to HbA1c levels only in patients with T1D. We found that all patients with T1D and HbA1c < 7.0% (53 mmol/mol) had adequate HL.

Currently, diabetes management, including its treatment is very complex requiring special HL skills related to numeracy as well to reading and comprehension, and both could be considered important tools to help patients to take appropriate health decisions. Many factors may influence HL such as age, years of school attendance, ethnicity, socioeconomic status and psychological distress.

Considering the general population, inadequate HL has been found to be very frequent worldwide [29, 33] and up to this moment, no method can be considered as the gold standard for its measurement. So far, several instruments were used for the assessment of HL in patients with diabetes, including the S-TOFHLA, used in the present study [32, 36]. It is important to note that most of the included studies were cross-sectional with insufficient or low level of evidence [26].

Our data showed that diabetes per se did not influence the results of S-TOFHLA, but glycemic control was influenced by the level of literacy, according to the scores obtained through the application of S-TOFHLA. However, this finding must be viewed with caution as well as its relation with the higher prevalence of inadequate HL among patients with T2D compared with patients having T1D, probably due to higher years of school attendance found in this latter group and the higher age found in patients with T2D. We do think that only future prospective studies could answer if diabetes, either T1D or T2D, has any impact on HL.

We have found that almost 18% of young patients with T1D were considered to have inadequate HL in comparison to 55.2% of the patients with T2D. The prevalence of inadequate HL, independent of the methods of measurement, among patients with T2D varies from 45.0% in Southeast of Brazil [44] to 65.9% in Northeast of Brazil [32] and from 10 to 69% in USA [25, 29, 36]. This fact shows that HL could be related to regional differences in education approaches, socioeconomic status, age, as well as to different characteristics of the diabetes care center included in the study. Similar to our data, in the majority of the above-mentioned studies, the most important variables associated with HL were years of school attendance and age. In the present study, performed with an admixed population, patients with T2D who self-reported as White, outperformed those who self-reported as non-White. A study conducted in the USA addressing only ethnic minorities did not find an association between HL, ethnicity and self-efficacy [37]. However, this topic is still controversial mainly due to different study designs and studied populations [29, 31, 32, 36, 39].

Few studies have been conducted exclusively with T1D or their caregivers [28, 30, 34, 38]. In our study, the prevalence of inadequate HL was 18% and it was lower than that reported in Pakistan, which was 67.2% [28] and United Kingdom (UK), which was 75% [30]. These conflicting results could be related to differences in educational level, age and socioeconomic status of the patients included in the studies as well as to different criteria used to define HL. In the present study, although we did not observe a correlation between HL and HbA1c, all the patients with HbA1c < 7.0% (53 mmol/mol) were literate, similarly to the findings from Pakistan [28]. Similar to our data concerning patients with T1D, a study that evaluated HL in children with T1D and their mothers did not find a correlation between HbA1c and HL of the children, but this was found regarding mothers’ HL [34]. Using the Diabetes Numeracy Test (DNT) in adolescents with T1D, a negative relationship was observed between the score and HbA1c [38]. The DNT include specific questions about sliding scale of insulin dosing, blood glucose monitoring, carbohydrate counting and exercise which are important tools for diabetes management. These questions are not addressed in S-TOFHLA. A study conducted in UK using the UK Adult Core Curriculum to asses literacy and numeracy skills showed an association between levels of HbA1c and numeracy scores but this association was not observed with literacy scores [30]. The above-mentioned facts point out that the evaluation of HL probably should be performed by different tools according to the type of diabetes as well as to the therapeutic regimens.

The major strength of our study is that we have performed the S-TOFHLA in all patients in the same context and in the same conditions within the diabetes center. Another point is that our population was from different self-reported color-race and socioeconomic background similar to the general Brazilian population.

We have also to address some limitations of our study. First, taking into account that our study was cross-sectional, we can only infer associations; so no causal relationship between the S-TOFHLA and glycemic control could be concluded. Secondly, some relevant points for achieving adequate glycemic control were not evaluated such as diabetes self-efficacy, psychological factors, poor family support and adherence to diabetes treatment. In a recent study conducted in Brazil with patients with T1D, depression was associated with high levels of HbA1c [45]. Thirdly, the lower number of patients with good glycemic control in our sample could have influenced our results. However, it is important to note that the latter fact was also found in other studies involving patients with diabetes in Brazil [14, 15, 45]. Finally, the lack of a control population matched for years of school attendance could have also compromised our results. However, few studies have been done with both populations, mainly with patients having T1D.

## Conclusion

In conclusion, in our study, a considerable number of patients with either T1D or T2D did not have adequate HL when evaluated by S-TOFHLA test. Overall, age and years of school attendance were the most important variables associated with better performance of the S-TOFHLA. In general, patients with T1D outperformed those with T2D. Although the majority of the patients did not present a good glycemic control, patients with T1D who had adequate HL reached more frequently HbA1c levels < 7% (53 mmol/mol). Finally, in addition to therapeutic regimens, an approach on diabetes management should also include patients’ HL evaluation together with psychological and social aspects aiming to improve glycemic control and avoiding short and long term adverse outcomes.

## Data Availability

Marilia Brito Gomes has full access to data and materials used in this research.

## Abbreviations

T1D: Type 1 diabetes
DCCT: Diabetes control and complications trial
EDIC: Epidemiology of diabetes interventions and complications
SMBG: Self-monitoring of blood glucose
BNHCS: Brazilian National Health Care System
BrazDiab1SG: Brazilian Type 1 Diabetes Study Group
HbA1c: Glycated hemoglobin
BMI: Body mass index
HPLC: High-performance liquid chromatography
CSII: Continuous subcutaneous insulin infusion
